# Diagnosis and Treatment of Anxiety Disorders in Autistic Patients: A Case Report

**DOI:** 10.1192/bjo.2024.664

**Published:** 2024-08-01

**Authors:** Anusha Govender

**Affiliations:** SWLSTG, London, United Kingdom

## Abstract

**Aims:**

Anxiety disorders are common in Autistic Spectrum Disorder (ASD) patients. There are limited resources dedicated to ASD and mental health services are not equipped to adapt assessment and treatment protocols to address their needs. Adaptations to diagnosis and treatment are discussed in a single case study of an autistic patient with anxiety disorders. In addition, effectiveness of providing adapted versus standard treatment is evaluated.

**Methods:**

This study describes a 45-year-old, single, employed male diagnosed as autistic at age 37. He was referred for a second course of Cognitive Behavioural Therapy (CBT) for anxiety disorders consisting of agoraphobia with panic; blood injury phobia; needle phobia; dental phobia; claustrophobia. The duration of symptoms was 35 years. The main impairments to functioning were inability to use public transport; attending healthcare appointments; going to public places; returning to office-based working.

Questionnaires routinely completed at assessment and end of treatment: Montgomery–Åsberg Depression Rating Scale (MADRS); Beck's Anxiety Inventory (BAI); Beck's Depression Inventory (BDI). Adapted treatment with CBT included an extended assessment which helped differentiate anxiety symptoms from ASD. Main CBT adaptations included development of skills for the patient to identify and express emotional experiences and thoughts with the focus on physical sensations and behaviour. Graded exposure items were linked to concrete aims or interests and structured to fit around the patient's routine daily activities. Clinical data was analysed and compared outcomes from the initial standard and subsequent adapted treatment.

**Results:**

The patient's response to the initial course of standard CBT showed a 14% increase in anxiety and 14% increase in symptoms of depression on self-rated measures. The subsequent adapted CBT showed a 31% improvement in anxiety and a 16% improvement in symptoms of depression on self-rated measures.

**Conclusion:**

This case report supports literature describing the need to adapt standard assessment and treatment to differentiate experiences related to ASD from discrete anxiety disorders, although there may be some overlap. The promising results support using adapted CBT to ensure appropriate treatment of anxiety disorders in autistic people.

